# Supplementary material for: Decoding the m6A epitranscriptomic landscape for biotechnological applications using a direct RNA sequencing approach
Source: Nat Commun. 2025 Jan 18;16:798. doi: 10.1038/s41467-025-56173-6 (PMC11742432; doi:10.1038/s41467-025-56173-6)
Supplement: Supplementary file 1 — Supplementary Information [file 41467_2025_56173_MOESM1_ESM.pdf]

Supplementary Information

Decoding the m^{6}A Epitranscriptomic Landscape for Biotechnological Applications Using A Direct RNA Sequencing Approach

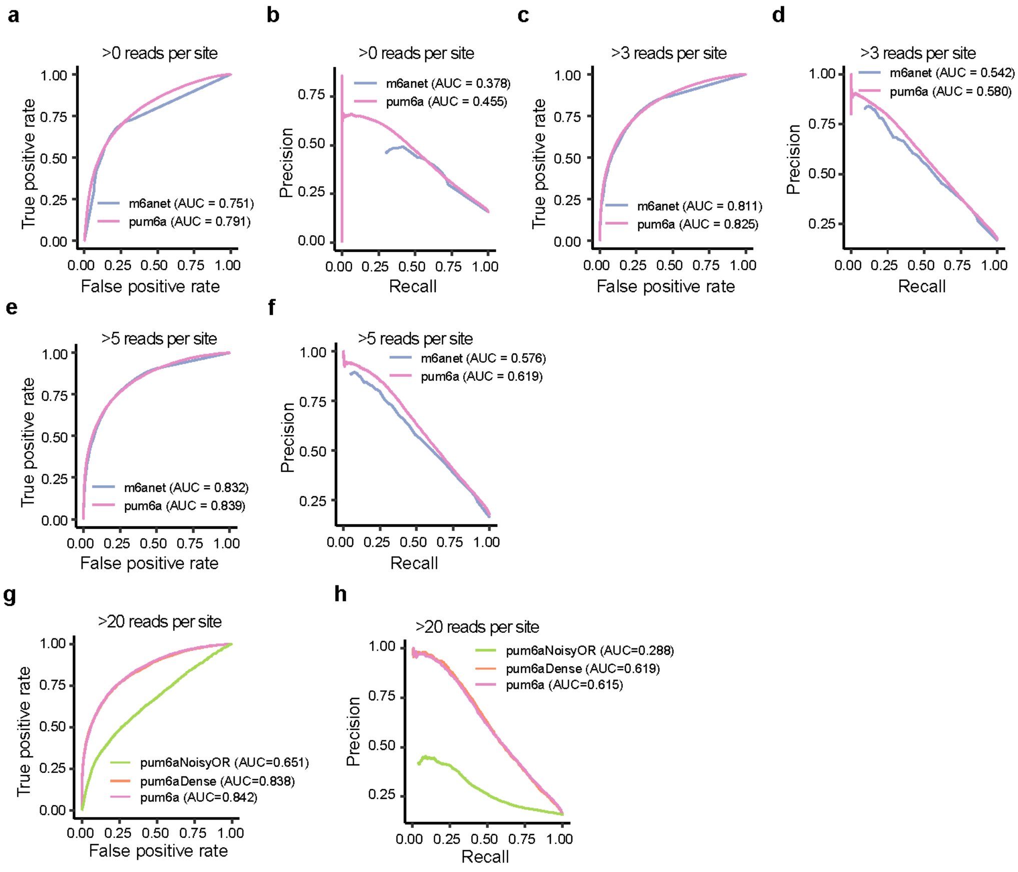

Supplementary Figure 1. Comparative performance of pum6a and m6anet under different thresholds. Comparison of pum6a's performance with m6anet using ROC (a), and PR curves (b) for datasets with at least 0 reads. Comparison of pum6a's performance with m6anet using ROC (c), and PR curves (d) for datasets with at least 3 reads. Comparison of pum6a's performance with m6anet using ROC (e), and PR curves (f) for datasets with at least 5 reads. Comparison of pum6a's performance with pum6aDense and pum6aNoisyOR using ROC (g) and PR curves (h) for datasets with at least 20 reads.

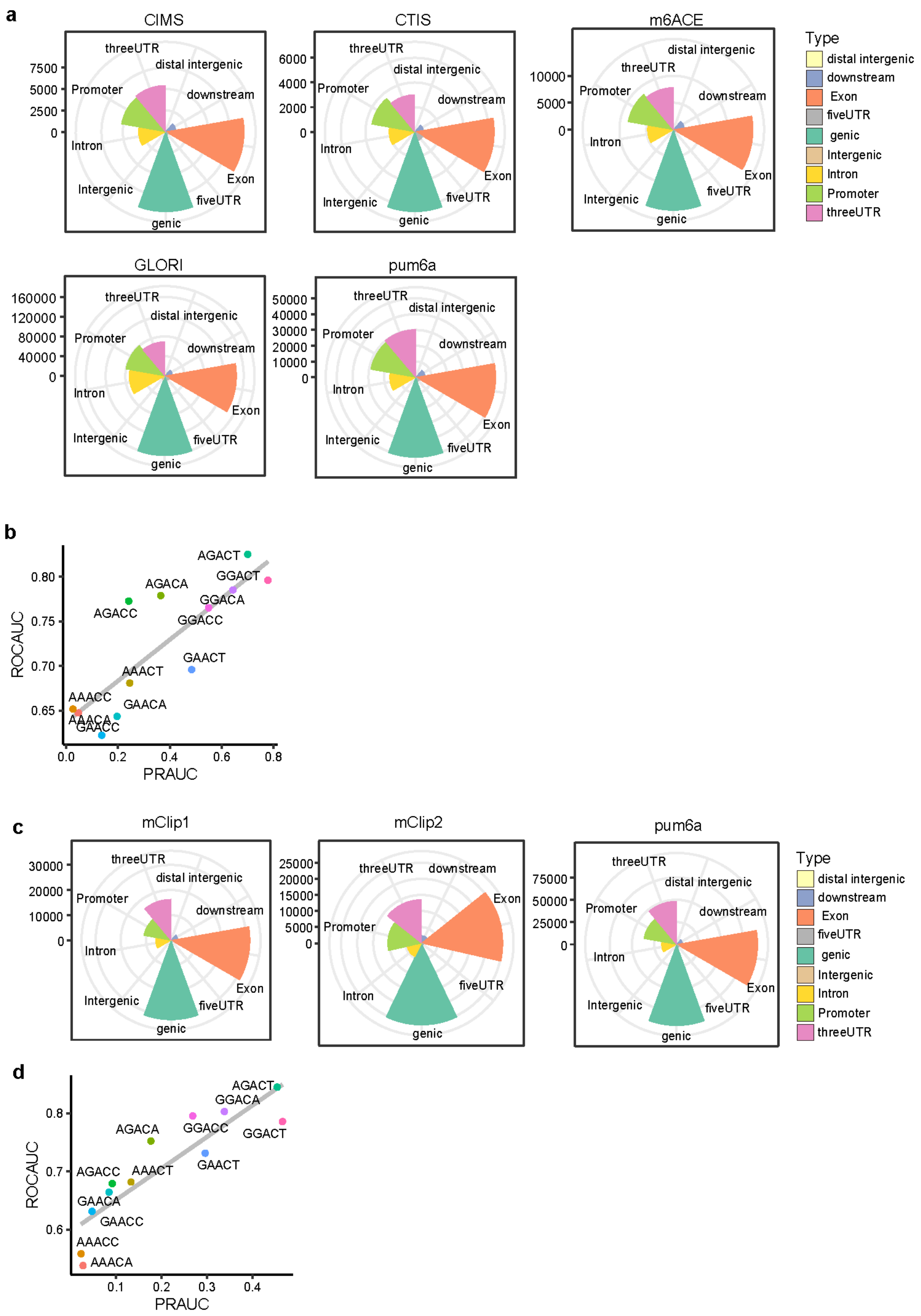

Supplementary Figure 2. Performance of pum6a in m^{6}A detection from ONT direct RNA sequencing data. Distribution of m^{6}A modification sites detected in HEK293T cells by different experimental protocols (a). Scatter plot of RRACH motif predictions by pum6a in HEK293T cells (b). Distribution of m^{6}A modification sites detected in mouse embryonic stem cells by different protocols (c). Scatter plot of RRACH motif predictions by pum6a in mouse embryonic stem cells (d).

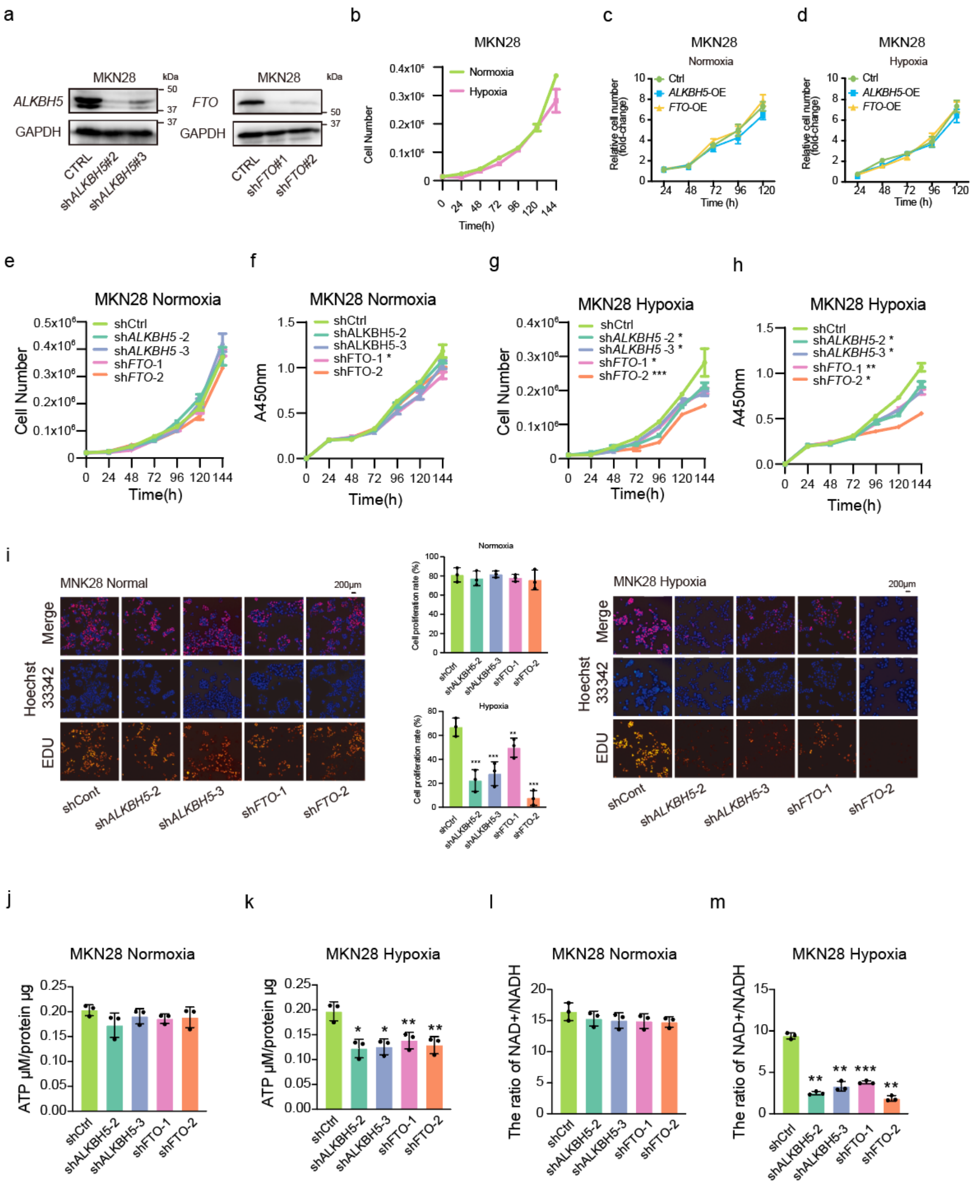

Supplementary Figure 3: Dynamic m^{6}A modification induced by hypoxia stress and m^{6}A demethylases in MKN28 cells. Western blot validation of m^{6}A demethylase (ALKBH5 and FTO) knockdown efficacy in MKN28 cells (a). Proliferation responses of MKN28 cells to hypoxic conditions, showing higher tolerance (b). Effects of ALKBH5 and FTO overexpression on MKN28 cell growth under normoxia (c) and hypoxia (d), quantified by cell count. Knockdown of ALKBH5 or FTO reduced cell number in MKN28 cells under hypoxia (g-h) but had no significant effects under normoxia (e-f). Knockdown of ALKBH5 or FTO significantly reduced cell proliferation in MKN28 cells under hypoxia, measured by EdU assay. Quantification of fold changes was performed using ImageJ (i). ATP levels were significantly reduced in MKN28 cells with FTO/ALKBH5 knockdown under hypoxia (j,k). NAD+ levels and NAD+/NADH ratio were significantly decreased in MKN28 cells following FTO/ALKBH5 depletion under hypoxia (l,m). Data are presented as the mean ± SD from three independent experiments. *$p < 0.05$, **$p < 0.01$, ***$p < 0.001$, by a two-way ANOVA (b-h) or t-test (i-m). Source data are provided as a Source Data file.

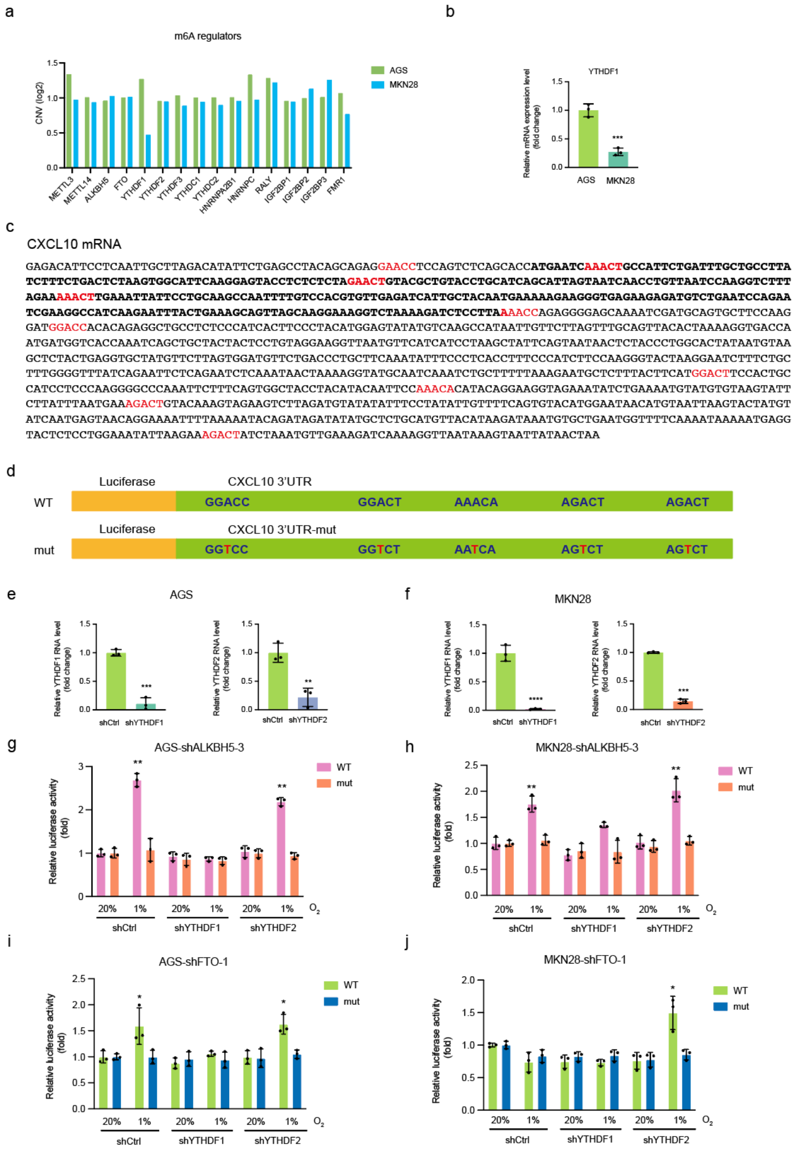

Supplementary Figure 4. Modulation of m⁶A modification in CXCL10 mRNA by m⁶A demethylases under hypoxia. Gene copy numbers of selected m⁶A modulators in AGS and MKN28 cells, including writers (METTL3, METTL14), erasers (ALKBH5, FTO), and readers (YTHDF1, YTHDF2, YTHDF3, YTHDC1, YTHDC2, HNRNPA2B1, HNRNPC, RALY, IGF2BP1, IGF2BP2, IGF2BP3 and FMR1) (a). Validation of YTHDF1 expression in AGS and MKN28 cells by qPCR (b). Full sequence of CXCL10 mRNA with predicted m⁶A sites identified by pum6a. Red highlights indicate potential m⁶A sites, bold sequences represent coding regions (c). Construction of luciferase reporter containing the CXCL10 3'UTR. Mutations were generated by replacing adenosine with thymine (d). Validation of shRNA-mediated knockdown of YTHDF1 and YTHDF2 in AGS (e) and MKN28 (f) cells by qPCR. Relative luciferase activity of wild-type and mutant CXCL10 3'UTR reporters in AGS (g) and MKN28 (h) cells, with ALKBH5, YTHDF1 and YTHDF2 knockdowns. Relative luciferase activity of wild-type and mutant CXCL10 3'UTR reporters in AGS (i) and MKN28 (j) cells with FTO, YTHDF1 and YTHDF2 knockdowns. Data are presented as the mean ± SD from three independent experiments. *$p < 0.05$, **$p < 0.01$, ***$p < 0.001$, by t-test. Source data are provided as a Source Data file.
