# Supplementary material for: Decoding the m6A epitranscriptomic landscape for biotechnological applications using a direct RNA sequencing approach
Source: Nat Commun. 2025 Jan 18;16:798. doi: 10.1038/s41467-025-56173-6 (PMC11742432; doi:10.1038/s41467-025-56173-6)
Supplement: Supplementary file 2 — Description of Additional Supplementary Files [file 41467_2025_56173_MOESM2_ESM.pdf]

Description of Additional Supplementary Files

File Name: Supplementary Data 1. Description: Performance of pum6a in the MNIST dataset and 20 anomaly detection datasets.

File Name: Supplementary Data 2. Description: List of m^6A modification sites in HEK293T cells identified by pum6a.

File Name: Supplementary Data 3. List of m^6A modification sites in mouse embryonic stem cells identified by pum6a.

File Name: Supplementary Data 4. Description: Gene counts for each AGS sample replicate from DRS data.

File Name: Supplementary Data 5. m^6A modification sites in AGS cells identified by pum6a.

File Name: Supplementary Data 6. Dynamic m^6A modified sites in AGS cells under different oxygen conditions identified by pum6a.

File Name: Supplementary Data 7. Enrichment analysis of m6A modification sites in AGS cells. Statistical analysis was performed using a two-sided hypergeometric test for pathway enrichment. Adjustments for multiple comparisons were made using the Benjamini-Hochberg method to control the false discovery rate.
