# Supplementary material for: Decoding the m6A epitranscriptomic landscape for biotechnological applications using a direct RNA sequencing approach
Source: Nat Commun. 2025 Jan 18;16:798. doi: 10.1038/s41467-025-56173-6 (PMC11742432; doi:10.1038/s41467-025-56173-6)
Supplement: Supplementary file 10 — Reporting Summary [file 41467_2025_56173_MOESM10_ESM.pdf]

nature portfolio

Corresponding author(s): Guohui Wan, Weiling He, Zhi Wang

Last updated by author(s): Dec 3, 2024

Reporting Summary

Nature Portfolio wishes to improve the reproducibility of the work that we publish. This form provides structure for consistency and transparency in reporting. For further information on Nature Portfolio policies, see our Editorial Policies and the Editorial Policy Checklist.

Statistics

For all statistical analyses, confirm that the following items are present in the figure legend, table legend, main text, or Methods section.

n/a	Confirmed	
☐	☒	The exact sample size (*n*) for each experimental group/condition, given as a discrete number and unit of measurement
☐	☒	A statement on whether measurements were taken from distinct samples or whether the same sample was measured repeatedly
☐	☒	The statistical test(s) used AND whether they are one- or two-sided<br>*Only common tests should be described solely by name; describe more complex techniques in the Methods section.*
☐	☒	A description of all covariates tested
☐	☒	A description of any assumptions or corrections, such as tests of normality and adjustment for multiple comparisons
☐	☒	A full description of the statistical parameters including central tendency (e.g. means) or other basic estimates (e.g. regression coefficient) AND variation (e.g. standard deviation) or associated estimates of uncertainty (e.g. confidence intervals)
☐	☒	For null hypothesis testing, the test statistic (e.g. *F*, *t*, *r*) with confidence intervals, effect sizes, degrees of freedom and *P* value noted<br>*Give P values as exact values whenever suitable.*
☒	☐	For Bayesian analysis, information on the choice of priors and Markov chain Monte Carlo settings
☒	☐	For hierarchical and complex designs, identification of the appropriate level for tests and full reporting of outcomes
☒	☐	Estimates of effect sizes (e.g. Cohen's *d*, Pearson's *r*), indicating how they were calculated

Our web collection on statistics for biologists contains articles on many of the points above.

Software and code

Policy information about availability of computer code

Data collection	https://github.com/liuchuwei/pum6a
Data analysis	The pum6a framework demonstrated superior performance in multi-instance anomaly detection when compared to several state-of-the-art methods, including PUMA and the Inexact Autoencoder (IAE), as well as other baselines like Random Forest and puIF (an unsupervised Isolation Forest with logistic regression). We evaluated pum6a across a range of datasets, including a modified MNIST image dataset and 20 well-established benchmarks for anomaly detection. To ensure robustness and generalizability, the model was tested using a stratified 5-fold cross-validation procedure, with varying label frequencies (10% to 50%), and a weighted Noisy-OR method for consistent interpretation.

For manuscripts utilizing custom algorithms or software that are central to the research but not yet described in published literature, software must be made available to editors and reviewers. We strongly encourage code deposition in a community repository (e.g. GitHub). See the Nature Portfolio guidelines for submitting code & software for further information.

Data

Policy information about availability of data

All manuscripts must include a data availability statement. This statement should provide the following information, where applicable:

- Accession codes, unique identifiers, or web links for publicly available datasets
- A description of any restrictions on data availability
- For clinical datasets or third party data, please ensure that the statement adheres to our policy

The HEK293T cell lines data were obtained from the Singapore Nanopore Expression Project through https://github.com/GoekeLab/sg-nex-data (ENA PRJEB44348). The mouse embryo data was downloaded from the Gene Expression Omnibus (GEO) database under accession number GSE195618. And the constructed data were downloaded from the GEO database under the accession code GSE124309 ("curlcakes"). The benchmark anomaly datasets were downloaded through the Link: https://github.com/Minqi824/ADBench/tree/main/datasets/Classical.

Research involving human participants, their data, or biological material

Policy information about studies with human participants or human data. See also policy information about sex, gender (identity/presentation), and sexual orientation and race, ethnicity and racism.

Reporting on sex and gender	NA
Reporting on race, ethnicity, or other socially relevant groupings	NA
Population characteristics	NA
Recruitment	NA
Ethics oversight	NA

Note that full information on the approval of the study protocol must also be provided in the manuscript.

Field-specific reporting

Please select the one below that is the best fit for your research. If you are not sure, read the appropriate sections before making your selection.

☒ Life sciences ☐ Behavioural & social sciences ☐ Ecological, evolutionary & environmental sciences

For a reference copy of the document with all sections, see nature.com/documents/nr-reporting-summary-flat.pdf

Life sciences study design

All studies must disclose on these points even when the disclosure is negative.

Sample size	Sample sizes were determined based on prior similar studies in RNA modification detection using nanopore sequencing. Statistical power calculations were not performed. We selected sample sizes that are commonly accepted in the field for direct RNA sequencing experiments, ensuring that the sample size was sufficient to capture variability in m6A modifications.
Data exclusions	No data were excluded from the analyses unless they failed quality control checks. The exclusion criteria were pre-established, focusing on samples with low sequencing quality or inadequate read coverage to maintain data reliability.
Replication	All experiments were performed in at least three biological replicates to ensure reproducibility. Results were consistent across replicates, supporting the reliability of our findings. We observed no discrepancies that could not be reproduced, indicating robustness in our methodology and analysis.
Randomization	Randomization was not applicable in this study, as all samples were treated under defined experimental conditions. Allocation into experimental groups was based on predetermined treatment conditions (normoxia and hypoxia for cell culture experiments) rather than random selection.
Blinding	Blinding was not conducted, as it was not relevant for this study. The analyses primarily involved automated data processing pipelines and bioinformatics tools, reducing the potential for subjective bias in interpretation.

Reporting for specific materials, systems and methods

We require information from authors about some types of materials, experimental systems and methods used in many studies. Here, indicate whether each material, system or method listed is relevant to your study. If you are not sure if a list item applies to your research, read the appropriate section before selecting a response.

Materials & experimental systems

n/a	Involved in the study	
☐	☒	Antibodies
☐	☒	Eukaryotic cell lines
☒	☐	Palaeontology and archaeology
☒	☐	Animals and other organisms
☒	☐	Clinical data
☒	☐	Dual use research of concern
☒	☐	Plants

Methods

n/a	Involved in the study	
☒	☐	ChIP-seq
☒	☐	Flow cytometry
☒	☐	MRI-based neuroimaging

Antibodies

Antibodies used	anti-ALKBH5, anti-FTO, anti-GAPDH
Validation	rabbit monoclonal anti-ALKBH5 (1:1000, A11684, ABclonal), rabbit monoclonal anti-FTO (1:1000, A3861, ABclonal), and anti-GAPDH (1:1000, 5147, Cell Signaling Technologies).

Eukaryotic cell lines

Policy information about cell lines and Sex and Gender in Research

Cell line source(s)	The eukaryotic cell lines used in this study were obtained from a reputable cell line repository ATCC. The cell lines utilized include AGS and MKN28. Both cell lines are derived from human gastric cancer cells.
Authentication	Authentication of the cell lines was performed through short tandem repeat (STR) profiling, ensuring that the cells matched the known STR profile of the respective cell line. Authentication checks were performed upon initial receipt and prior to conducting major experiments to confirm cell line identity.
Mycoplasma contamination	All cell lines used in this study were tested for mycoplasma contamination before and during the study period using a PCR-based mycoplasma detection kit. The results confirmed that all cell lines were free of mycoplasma contamination.
Commonly misidentified lines (See ICLAC register)	NA

Plants

Seed stocks	NA
Novel plant genotypes	NA
Authentication	NA
